# Metagenomic next-generation sequencing in diagnosing *Pneumocystis jirovecii* pneumonia: A case report

**DOI:** 10.1515/biol-2022-0094

**Published:** 2022-08-17

**Authors:** Yuan Zhang, Zhaoshang Zeng, Fenghui Li, Zhiyun Peng, Han Xia, Yunyi Zeng, Haimin Chen, Yingjing Wang, Weining Xie, Yanhua Zhang, Zhongxiang Tang

**Affiliations:** Department of Intensive Care Unit, Guangdong Provincial Hospital of Integrated Traditional Chinese and Western Medicine, Foshan 528200, China; Department of Scientific Affairs, Hugobiotech Co., Ltd, Beijing 100176, China; Department of Infectious Disease, Guangdong Provincial Hospital of Integrated Traditional Chinese and Western Medicine, Foshan 528200, China; Department of Nutriology, Guangdong Provincial Hospital of Integrated Traditional Chinese and Western Medicine, Foshan 528200, China

**Keywords:** *Pneumocystis jirovecii* pneumonia, rheumatoid arthritis, diagnosis, metagenomic next-generation sequencing, case report

## Abstract

It remains a huge challenge for clinicians to diagnose *Pneumocystis jirovecii* pneumonia (PJP) by a conventional method, which leads to delay in diagnosing PJP, accounting for higher mortality in patients with rheumatoid arthritis (RA). A 69-year-old woman, who suffered from RA for years, developed acute respiratory failure. The computed tomography scan showed diffused effusion and ground glass opacity in both lungs, which could not be differentiated from interstitial pneumonia. Metagenomic next-generation sequencing (mNGS) revealed *P. jirovecii* in both serum and bronchoalveolar lavage fluid with reads per million (RPM) of 17 and 437, while other diagnostic tests did not detect any pathogenic microorganism. The results were verified by quantitative polymerase chain reaction (mtSSU region) against the same samples. The DNA RPM of *P. jirovecii* declined notably after treatment with trimethoprim/sulfamethoxazole. The patient was discharged without treatment and finally passed away. This case fully highlights the sensitivity of mNGS in early diagnosis of PJP, which is of great significance for prognosis and treatment. Nonetheless, the clinical application of mNGS is worth further standardization and normalization.

## 1 Background

*Pneumocystis jirovecii* pneumonia (PJP) is an opportunistic, life-threatening infection. *P. jirovecii* is more likely to infect the immunocompromised population, such as patients with human immunodeficiency virus (HIV). Therefore, PJP is known as the most common complication of patients with HIV. Owing to the application of highly active antiretroviral therapy and routine prophylaxis of PJP, the incidence of PJP in patients with HIV declined significantly [1]. However, a substantial increase in prevalence was also observed among the non-HIV population [2], including patients with hematological and solid malignancies, autoimmune disorders, inflammatory diseases, and hematological and solid organ transplantations. Besides the difference in incidence, clinical, radiological, and biological presentations also differ between HIV-positive and HIV-negative individuals with different immunodeficiency profiles [3]. HIV-infected patients usually develop a subacute course of the disease, while dyspnea occurs more quickly and progresses more rapidly in non-HIV-infected immunocompromised patients, with a higher risk of respiratory failure and mortality [4]. It is thought to be related to the different immune statuses. It seems that non-HIV patients probably have a more effective immune system with greater intensity of the inflammatory response, resulting in a bleaker prognosis, while the ones in patients with HIV do not function well with low CD4^+^ lymphocyte counts [5]. Currently, the mortality rate in HIV-infected patients ranges from 10 to 20%, whereas in non-HIV-infected patients is higher, ranging from 30 to 60% [6]. Delay in PJP diagnosis may account for higher mortality.

Unfortunately, the nonspecific symptoms, including loss of appetite, shortness of breath, diarrhea, fatigue, and a dry cough, fever or not, make it difficult for clinicians to diagnose the disease accurately, especially in patients with rheumatoid arthritis (RA). In addition, because it is hard to obtain definitive cultural evidence of *P. jirovecii* [7], the microscopic demonstration of the organisms in respiratory specimens has been considered the golden standard for the diagnosis of PJP [8]. However, its sensitivity cannot always satisfy the need of clinicians. The biomarkers such as serum beta-d-glucan and serum lactate dehydrogenase seem to be sensitive enough but are with a high false positive rate [9]. Based on specific DNA sequences, polymerase chain reaction (PCR) appears to be an optimal method to diagnose PJP. Nevertheless, it is difficult to distinguish *P. jirovecii* colonization from infection [10]. Hence, a diagnosis of PJP should be discreetly made based on all kinds of clinical data, including PCR, serum beta-d-glucan, serum lactate dehydrogenase, clinical presentations, and imaging features.

Currently, with the development of molecular biology, metagenomic next-generation sequencing (mNGS) provides an alternative approach to assist in early clinical diagnosis with all microbes in a sample identified [11,12]. In this case, the pathogen detection from serum and bronchoalveolar lavage fluid (BALF) was performed based on mNGS platform, and this suspected diagnosis was confirmed by quantitative polymerase chain reaction (qPCR).

## 2 Case presentation

A 69-year-old woman with a slight shortness of breath was admitted to our hospital (December 26th, 2020), complaining of weakness in her arms and legs for the last week. Almost all the typical symptoms, including fever, cough, rhinobyon, previous asthma, COPD, and poor working or living environment conditions, such as smoking and contact with pets and poultry, were denied by herself. Additionally, she did not contact suspected COVID-19 cases, with negative PCR results for COVID-19 (Table 1). She suffered from RA for 30 years, taking a long-term course of steroids. Recently, as the symptoms progressed, the therapeutic schedule was changed to include methotrexate, leflunomide, and prednisone. Given the suspicion of peripheral neuropathy or anemia, she was treated with neurotrophic agents. Upon admission to our hospital, the physical examination found a body temperature of 36.2°C, blood pressure of 142/83 mmHg, pulse of 105 beats per minute, and a respiration rate of 20 per minute. Fine moist rales appeared in the lower lobes of the lungs. She presented muscle strength of grade 5 for upper limbs and grade 3 for lower ones.

**Table 1:** The results of conventional methods and mNGS of this patient

Time of sample collection	Time of test report	Diagnostic methods	Results
Dec. 26	Dec. 26	PCR of COVID-19	Negative
	Dec. 29	Sputum/urine/blood cultures	Negative
Dec. 28	Dec. 30	BALF culture	Negative
	Dec. 29	BALF smear	Gram-negative
	Dec. 29	BALF GeneXpert MTB	Negative
	Dec. 29	BALF GM test	Negative
	Dec. 29	BALF mNGS	*P. jirovecii*
	Dec. 29	Blood mNGS	*P. jirovecii*
	Jan. 3	Bone marrow biopsy	Negative
Dec. 29	Dec. 30	qPCR of *P. jirovecii*	Positive
Jan. 3	Jan. 4	BALF mNGS	*P. jirovecii*
	Jan. 4	Blood mNGS	*P. jirovecii*
Jan. 12	Jan. 13	BALF mNGS	*Acinetobacter baumannii*
	Jan. 13	Blood mNGS	*A. baumannii*
Jan. 18	Jan. 19	BALF mNGS	*K. pneumoniae*
	Jan. 19	Blood mNGS	*K. pneumoniae*

**Figure 1:**
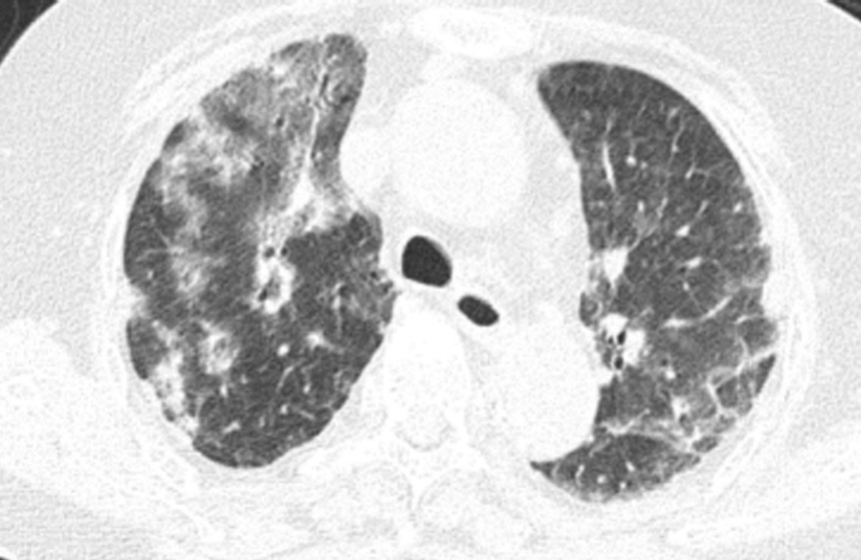
The chest images of this patient. The CT scan showed diffused effusion and GGO in both lungs.

Her condition deteriorated at night, with sudden polypnea and low-grade fever. Due to hypoxemia (pO_2_ 68 mmHg, pCO_2_ 21.6 mmHg), the patient was given oxygen therapy with a mask and atomization inhalation. The computed tomography (CT) scan showed diffused effusion and ground glass opacity (GGO) in both lungs (Figure 1). Other laboratory tests revealed three series decrease in peripheral blood, especially in lymphocyte counts, abnormal renal function with creatinine value of 230.5 μmol/L, and blood urea nitrogen value of 16.6 mmol/L.

Given the rapid disease progression, the patient was transferred to our intensive care units for further treatment at midnight on December 28th. She was sober but extremely fatigued, breathless, weak in all limbs, with a mild fever. The vital signs indicated a heart rate of 138 per minute, saturation of pulse oxygen (SpO_2_) of 85% on oxygen therapy with mask, and respiration rate of 31 per minute. Based on all the clinical symptoms and physical examinations, the diagnosis of acute respiratory failure was established. Intubation and mechanical ventilation were performed. At the same time, invasive blood pressure showed 66/42 mmHg, with sequential organ failure assessment score of 18. So, she was considered to be in the status of septic shock. Fluid resuscitation and vasoactive drugs were given following the 1-h bundle principles. To obtain etiological specimens, she underwent electric bronchoscopy with BALF collection. The BALF was tested through various methods, including culture, smear, GeneXpert MTB, and galactomannan test. Considering that the patient was highly suspected of opportunistic infections, such as PJP or cytomegalovirus infection, the mNGS for pathogen detection from serum and BALF were carried out immediately. Meanwhile, she was given oral trimethoprim/sulfamethoxazole (TMP/SMX, 320/1,600 mg q.i.d.) and intravenous acyclovir. Besides, other bacterial infections could not be excluded because of increasing inflammatory biomarkers, and she was given broad-spectrum antimicrobial therapy. Continuous renal replacement therapy was also performed for acute kidney injury.

**Figure 2:**
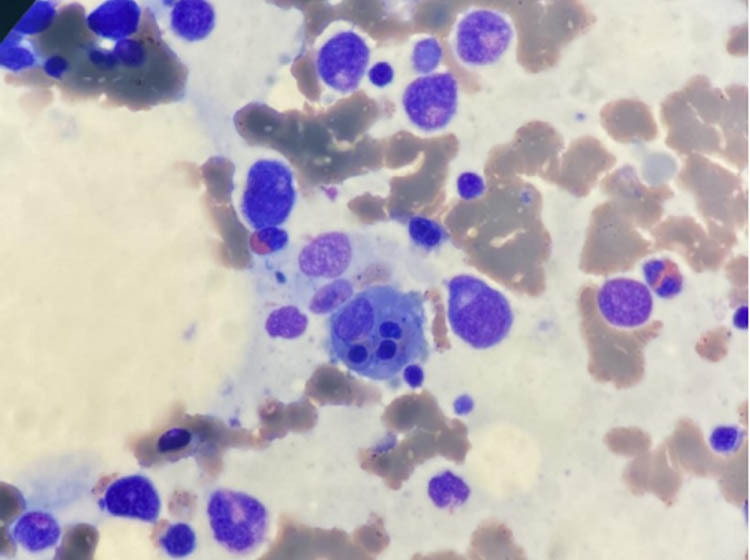
The bone marrow biopsy result of the patient. Hemophagocyte was detected in bone marrow smear.

Bone marrow biopsy was carried out on the second day of hospitalization (Day 2). The results revealed that the hemophagocyte was detected in the smear (Figure 2). It was inferred that the condition could be associated with severe infection.

**Figure 3:**
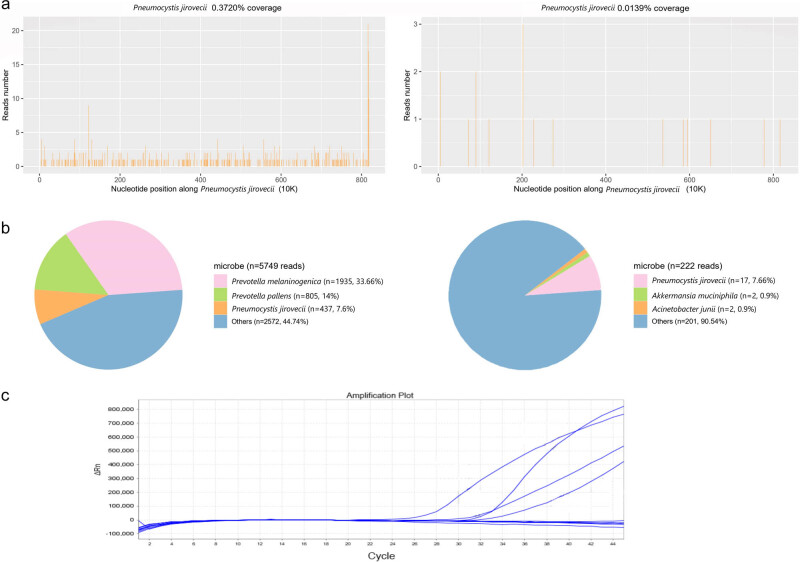
The results of mNGS and qPCR: (a) coverage and reads number of detected *P. jirovecii* by BALF-mNGS on December 28th, (b) coverage and reads number of detected *P. jirovecii* by blood-mNGS on December 28th, and (c) qPCR results of mtSSU region of *P. jirovecii*, four out of the eight samples were positive.

On the third day, the sputum, urine, and blood cultures returned with negative results (Table 1). Gram stain of the BALF revealed many Gram-negative organisms. mNGS revealed *P. jirovecii* in both serum and BALF with reads per million (RPM) of 17 and 437 (Figure 3), while other diagnostic tests did not detect any pathogenic microorganism. The mtSSU region of *P. jirovecii* was subsequently detected by qPCR using the same samples (Figure 3). Four out of the eight samples gave positive results. The PCR products were sequenced by Sanger and finally identified as *P. jirovecii*, confirming the mNGS results. The results were verified by qPCR using the same samples. Accordingly, the patient was treated with TMP/SMX, caspofungin, and thymalfasin to boost immunity.

**Figure 4:**
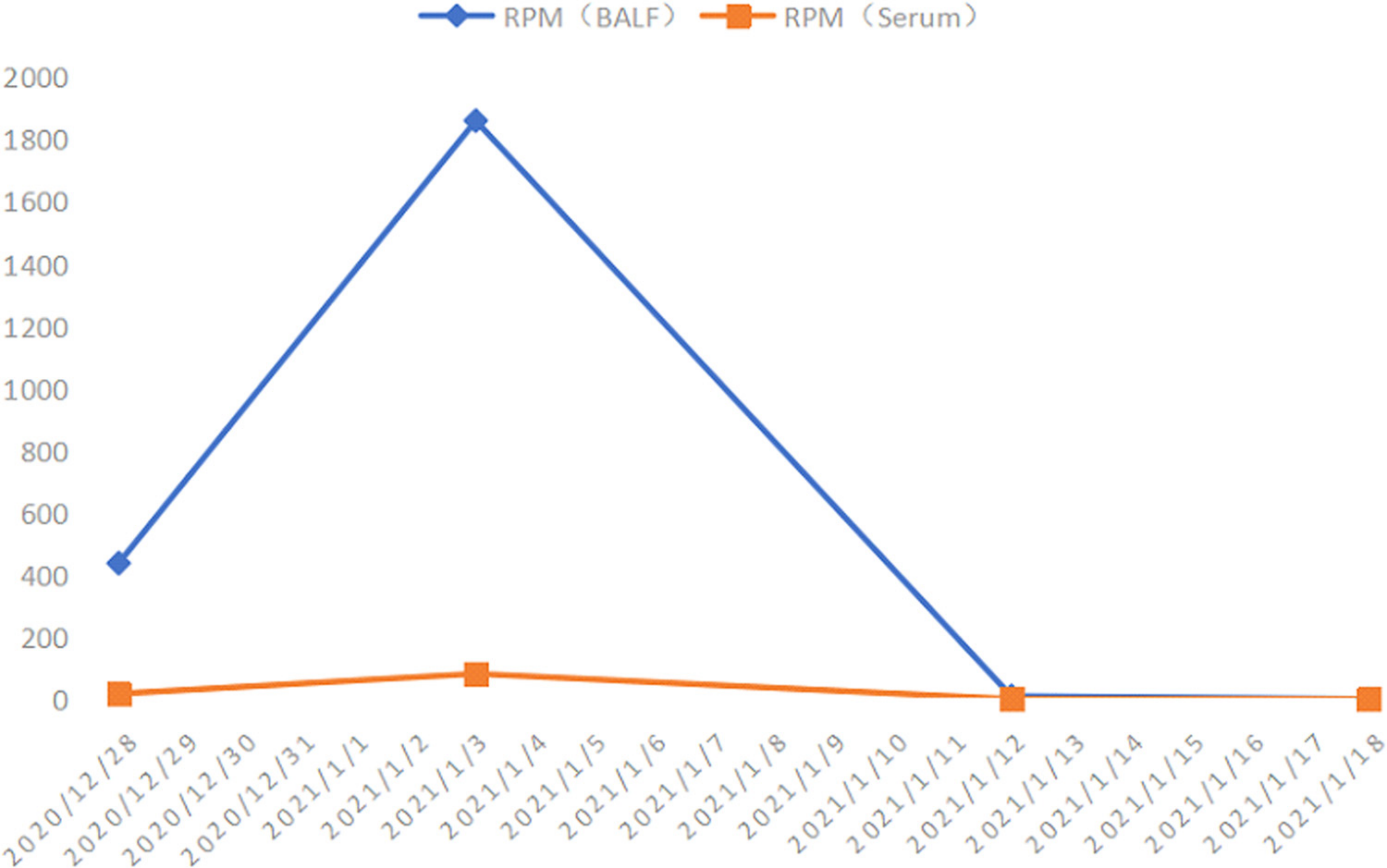
Change of *P. jirovecii* DNA reads in BALF and serum. During the 21-day treatment, the DNA reads of *P. jirovecii* by mNGS using both serum and BALF disclosed a dramatic increase since the medication of TMP/SMX, then declined gradually.

Corticosteroids could suppress the acute inflammatory process [13]. However, the X-ray showed that effusion did not decrease as expected. Despite attempts at optimizing gas exchange by prone position ventilation for nearly a week, the arterial blood gas analysis indicated that pO_2_/FiO_2_ ratio was still below 100 mmHg, which made it necessary to take a rescue extracorporeal membrane oxygenation (ECMO) therapy. Mechanical ventilation was switched to an ultra-protective strategy using pressure control ventilation. On the 17th day of admission, a chest X-ray revealed an obvious decrease in exudation. She was successfully de-cannulated after 9 days of V-V ECMO support. During the 21-day treatment, the DNA reads of *P. jirovecii* by mNGS using both serum and BALF disclosed a dramatic increase since the medication of TMP/SMX, then declined gradually (Figure 4).

Unfortunately, acute hepatic failure occurred. Plasma bilirubin levels remained high despite the support of plasma exchange and resin plasma perfusion adsorption. mNGS of serum and BALF detected *Acinetobacter baumannii* on January 13th and *Klebsiella pneumoniae* on January 19th (Table 1), indicating nosocomial infection. Drug susceptibility tests revealed multidrug-resistant bacteria infection (*A. baumannii* resistant to quinolones, β-lactam antibiotics, sulfonamides, and aminoglycosides; *K. pneumoniae* resistant to quinolones, β-lactam antibiotics, and sulfonamides), leading to prolonged stay in hospital. Finally, the patient died on the 29th day of admission.


**Informed consent:** Informed consent has been obtained from all individuals included in this study.
**Ethical approval:** The research related to human use has been complied with all the relevant national regulations, institutional policies and in accordance with the tenets of the Helsinki Declaration, and has been approved by the ethical review committee of Guangdong Provincial Hospital of Integrated Traditional Chinese and Western Medicine.

## 3 Discussion and conclusions

Over the past decade, medical technology has advanced considerably, as well as the increasing cognition about autoimmune diseases, contributing to the progress of immunosuppressive therapy, including corticosteroids, molecular-targeted agents, and methotrexate. However, in the meantime, immunosuppressive therapy has also exposed the patients to the risk of PJP, especially for patients with RA [14]. Although PJP among RA patients is rare, it would be fatal. Thus, the key to the treatment and prognosis of PJP is timely diagnosis.

However, it remains challenging for clinicians to distinguish PJP from other infectious diseases due to its nonspecific symptoms and the insufficient sensitivity and specificity of traditional diagnostic methods, such as smears and stains of sputum and BALF, the serum 1,3-β-d-glucan [15]. To overcome the limitations, molecular biology-based methods, such as conventional PCR, have been developed rapidly, with high sensitivity in detecting *P. jirovecii* DNA [10]. However, it may have high false-positive rates and low positive predictive values.

mNGS provides a reliable approach to clinical decision-making with higher sensitivity and specificity. Almost all known or unknown microorganisms could be detected without hypothesis or bias. The process of mNGS usually takes less than 2 days, enabling clinicians to confirm the pathogen as soon as possible. In addition, the drug resistance gene of pathogens could be identified by mNGS for directing medication regimen and treatment [16].

In recent years, clinical practice consensus for the application of mNGS was reached in China. It is recommended to be used for infections of unknown origin especially in critical ill patients or immunodeficient ones [17]. The negative results can also provide meaningful information for immunocompromised patients to exclude the likelihood of infection.

Nevertheless, there are still some obstacles in the application of mNGS, such as the high cost and the lack of standardization. Another potential drawback of mNGS is that most reads are derived from human hosts. Accordingly, the pathogen-derived reads account for only a small percentage, resulting in decreased analytic sensibility [18]. Measures are available to mitigate false negatives, such as host depletion methods or microbial enrichment [19]. However, microbial enrichment methods may decrease analytic specificity and add bias to the diagnosis [19].

It remains a huge challenge for clinicians to interpret the mNGS results accurately. It is difficult to differentiate pathogens from colonization or contamination for nonsterile specimens, such as BALF, stool, or polymicrobial abscesses. Quantitative mNGS methods are applied to aid the differentiation of pathogens, but further investigation is needed [20]. A previous study indicated that the optimal cut-off reads a number of common pathogens contributing to the development of sepsis, including *A. baumannii*, *Pseudomonas aeruginosa*, and *K. pneumoniae*, was relatively high [21]. To a certain extent, the definite cut-off value for each pathogen facilitates the data interpretation of mNGS, but requires more research.

Interestingly, we could find a significant rise of RPM detected for *P. jirovecii* in BALF and serum specimens after 1-week treatment of TMP/SMX on January 3rd. It is well known that the cyst wall of *P. jirovecii* is so thick that it cannot be damaged easily. This abnormal rise was believed to be associated with the lysis of the cyst wall after targeted therapy, releasing DNA sequences. On the other hand, the decline of DNA sequences was in accord with improved clinical manifestations and imaging signs. In addition, the existence of *P. jirovecii* was verified by qPCR.

In general, we presented a case with PJP, an elderly female patient with a history of RA who has been taking corticosteroids and immunosuppressors for years. mNGS detected *P. jirovecii* in BALF and serum samples verified by qPCR, while traditional diagnostic methods failed to find any pathogens. This case fully highlighted the sensitivity of mNGS in the timely diagnosis of PJP, which is of great significance for the treatment and prognosis. In the application of NGS is required standardization and validation according to international rules.
